# Balancing the Load: How Optimal Forces Shape the Longevity and Stability of Orthodontic Mini-Implants

**DOI:** 10.3390/dj13020071

**Published:** 2025-02-05

**Authors:** Tinela Panaite, Carmen Savin, Nicolae Daniel Olteanu, Cristian Liviu Romanec, Raluca-Maria Vieriu, Carina Balcos, Alice Chehab, Irina Nicoleta Zetu

**Affiliations:** Department of Oral and Maxillofacial Surgery, Faculty of Dental Medicine, “Grigore. T. Popa” University of Medicine and Pharmacy, 16 Universitatii Str., 700115 Iasi, Romania; tinela-panaite@umfiasi.ro (T.P.); carmen.savin@umfiasi.ro (C.S.); daniel.olteanu@umfiasi.ro (N.D.O.); raluca-maria.vieriu@umfiasi.ro (R.-M.V.); carina.balcos@umfiasi.ro (C.B.); alice-chehab@umfiasi.ro (A.C.); irina.zetu@umfiasi.ro (I.N.Z.)

**Keywords:** cortical bone, finite element method (FEM), fracture, mini-implant, orthodontic forces, stress distribution

## Abstract

**Objective**: This study aims to investigate the mechanical behavior of titanium (Ti6Al4V) mini-implants (MIs) under varying orthodontic forces using finite element analysis (FEA) and to evaluate their performance and durability under realistic clinical conditions. Optimal orthodontic forces significantly influence the structural integrity and functional longevity of MIs while minimizing adverse effects on surrounding bone tissues. **Materials and Methods**: A commercially available MI (diameter: 2.0 mm, length: 12 mm) was modeled using FEA. The mandible geometry was obtained using computed tomography (CT) scanning, reconstructed in 3D using SpaceClaim software 2023.1, and discretized into 10-node tetrahedral elements in ANSYS Workbench. Material properties were assigned based on the existing literature, and the implant–bone interaction was simulated using a nonlinear frictional contact model. Orthodontic forces of 2 N and 10 N, inclined at 30°, were applied to simulate clinical loading conditions. Total displacement, von Mises stresses, equivalent strains, fatigue life, and safety factors were analyzed to assess the implant’s mechanical performance. **Results**: At 2 N, the MI demonstrated minimal displacement (0.0328 mm) and sustained approximately 445,000 cycles under safe fatigue loading conditions, with a safety factor of 4.8369. At 10 N, the implant’s lifespan was drastically reduced to 1546 cycles, with significantly elevated stress (6.468 × 10^5^ MPa) and strain concentrations, indicating heightened risks of mechanical failure and bone damage. The findings revealed the critical threshold beyond which orthodontic forces compromise implant stability and peri-implant bone health. **Conclusions**: This study confirms that maintaining orthodontic forces within an optimal range, approximately 2 N, is essential to prolong MI lifespan and preserve bone integrity. Excessive forces, such as 10 N, lead to a rapid decline in durability and increased risks of failure, emphasizing the need for calibrated force application in clinical practice. These insights provide valuable guidance for enhancing MI performance and optimizing orthodontic treatment outcomes.

## 1. Introduction

Orthodontic MIs have gained widespread use as temporary anchorage devices (TADs) due to their ability to provide stable support during orthodontic treatments. Despite their advantages, the failure rates of MIs remain a concern, with reports ranging from 5% to 30%, influenced by factors such as placement site, patient characteristics, and loading conditions [1,2,3,4]. These failures are predominantly attributed to inadequate primary stability, which is important for maintaining the mechanical engagement between the MI and the surrounding bone under orthodontic forces [5,6]. One of the primary determinants of primary stability is the cortical bone thickness at the insertion site. Research shows that the anterior palatal region offers higher success rates compared to the buccal or interradicular spaces [7]. However, the influence of cortical bone thickness on the success of MIs, especially under varying orthodontic forces, remains underexplored. This gap in understanding is critical, as insufficient cortical bone support can lead to stress concentrations, microfractures, and MI loosening, thereby compromising treatment outcomes [8,9]. Cortical bone thickness plays a pivotal role in distributing mechanical stress during orthodontic tooth movement. While studies have demonstrated increased stress in thin cortical bone regions under orthodontic forces, its specific impact on MI stability and bone remodeling remains unclear [8]. This is particularly relevant in the context of TADs, where the interaction among implant design, insertion angle, and bone quality must be optimized to ensure mechanical stability [10,11,12]. Recent advances in orthodontic research, including finite element analysis (FEA), 3D simulations, and material innovations, provide new opportunities to refine MI design and placement techniques [13,14,15,16,17,18]. These tools allow for precise modeling of stress–strain distributions and patient-specific treatment planning, improving the predictability and efficacy of orthodontic interventions. For example, studies have shown that oblique insertion angles reduce stress on cortical bone, potentially lowering failure rates [4,19]. Additionally, the use of titanium alloys with properties closely matching bone has been found to minimize stress-shielding effects, further enhancing implant stability [20,21]. Despite these advancements, there is a lack of comprehensive studies addressing the interplay among cortical bone thickness, orthodontic forces, and MI stability. This study aims to bridge this gap by systematically investigating how variations in cortical bone thickness influence stress distribution and mechanical stability in MI. By leveraging tools such as FEA, this research seeks to provide evidence-based guidelines for optimizing MI placement and design, ultimately advancing current orthodontic practices. Optimal orthodontic forces significantly influence the structural integrity and functional longevity of MIs while minimizing adverse effects on surrounding bone tissues.

## 2. Materials and Methods

### 2.1. Geometric Modeling

**Figure 1:**
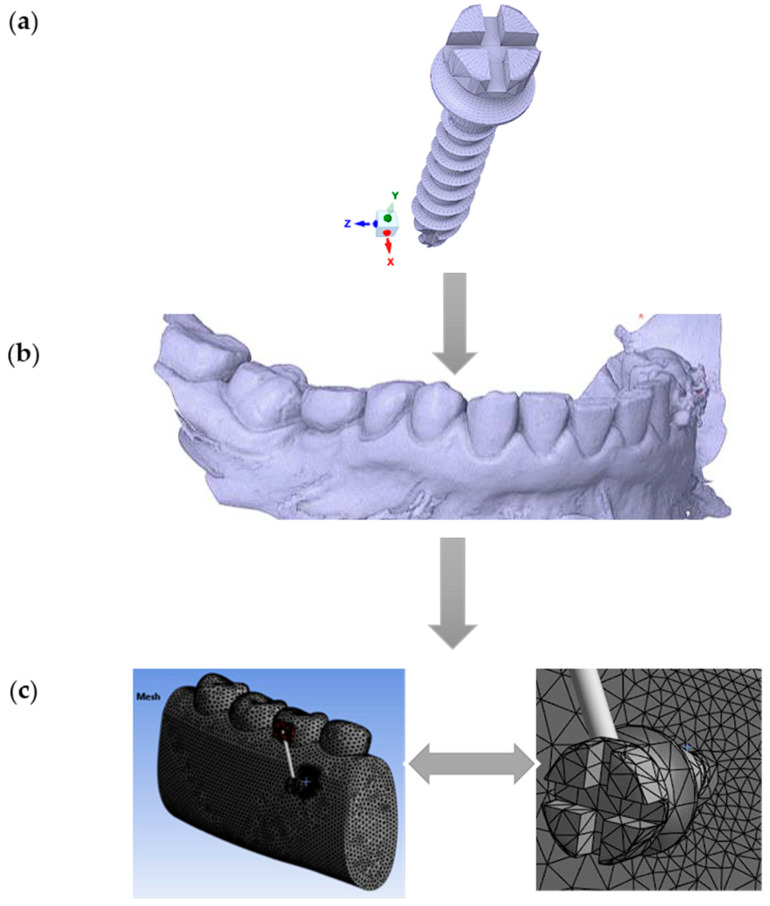
Workflow for finite element modeling of an orthodontic MI and adjacent bone structures: (**a**) Commercially available titanium MI, diameter 2 mm, was modeled using the finite element method; (**b**) Geometry of the mandible: CT scan image in STL format; (**c**) The global 3D geometric model discretized into finite elements (left) and the zoomed-in discretized model at the location of interest (right, MI and the adjacent orthodontic anchorage area).

In this study, a commercially available titanium (Ti6Al4V) MI (Jeil Medical Corporation, Seoul, South Korea), with a diameter of 2.0 mm and a length of 12 mm, was modeled using the finite element method (FEM) (Figure 1a). The mandible’s geometry was obtained through scanning, and the captured computed tomography (CT) (DEXIS, Biberach, Germany) images were digitized (Figure 1b). Three-dimensional solid models of the MI and mandible were then reconstructed and assembled using the commercial computer-aided design (CAD) software SpaceClaim 2023.1 (Figure 1c). 

### 2.2. Simulation Parameters

The insertion point of the MI was located in the space between the premolar and molar. The entire model was imported into the finite element analysis software (version 2021; ANSYS, Inc., Canonsburg, PA, USA) and discretized using automated 10-node tetrahedral structural elements. The bones, teeth, periodontal ligament, and MI were modeled as linear elastic, homogeneous, and isotropic materials. The mechanical properties of these materials were based on data from the existing literature (Table 1). The interfaces between the teeth and the periodontal ligament were assumed to be bonded, while a frictional contact (friction coefficient = 0) was assumed between the bone and the MI.

A force of 2 N and an additional 10 N force were applied at a 30° angle relative to the vertical axis (Y) to simulate real-world orthodontic conditions. These forces were directed from the MI toward the molar via the connector tube, accurately representing the biomechanics involved in a molar intrusion scenario with skeletal anchorage. This angled application reflects clinical load transfer during orthodontic treatments and allows for a realistic assessment of stress and strain distributions within the periodontal ligament, alveolar bone, and surrounding tissues.

### 2.3. Technical Details of FEA Modeling

Table 2 presents the parameters for finite element analysis (FEA). This table outlines the parameters of the finite element analysis model used in the specified study. Below is a brief description of each parameter:

1. Discretization (nodes/elements): This indicates the number of nodes and elements used in the FEA model. The higher the number of nodes and elements is, the more detailed the geometric representation of the object and its interactions.
2. Element: This refers to the type of finite elements used in the model, in this case, 10-node tetrahedrons. These elements are used to discretize the geometry of the object and calculate the structural response.
3. Software: This refers to the analysis software used to perform the finite element simulations. In this case, ANSYS is the specified software.
4. Material model: This describes the material properties used in the model. The specified model is isotropic, homogeneous, and linear. Thus, the material properties do not vary depending on direction or position. In addition, the material is uniform, and its behavior is linear under load.
5. Contact model: This describes how the interactions between different parts of the model are simulated. It mentions the use of nonlinear friction for the interaction between the MI and the bone, as well as linear bonding for other interactions.
6. Loading: This specifies the loads applied to the model. The loading is oblique, with values between 2 N and 10 N.
7. MI type: This describes the characteristics of the MIs used in the model, including dimensions and thread type.
8. Boundary conditions: This specifies the boundary conditions applied to the model. In this case, 100% osseointegration is mentioned, indicating that the MI is fully integrated into the bone and there is no relative movement between them.

## 3. Results

The structural static analysis was performed considering that the structure is an undamped system, ignoring any material behavior inhomogeneities. Stiffness was specified using the elastic and isotropic material model. The results obtained for the loading scenario with 2 N for the MI are presented below. It is inserted at a depth of 7 mm into the bone, and the orthodontic force is inclined at 30° relative to the vertical axis (Y). The MI is placed normal to the surface (at 90° to the cortical and spongy bone).

### 3.1. Determinations: Total Deformations, von Mises Equivalent Stresses, and Equivalent Strains

**Figure 2:**
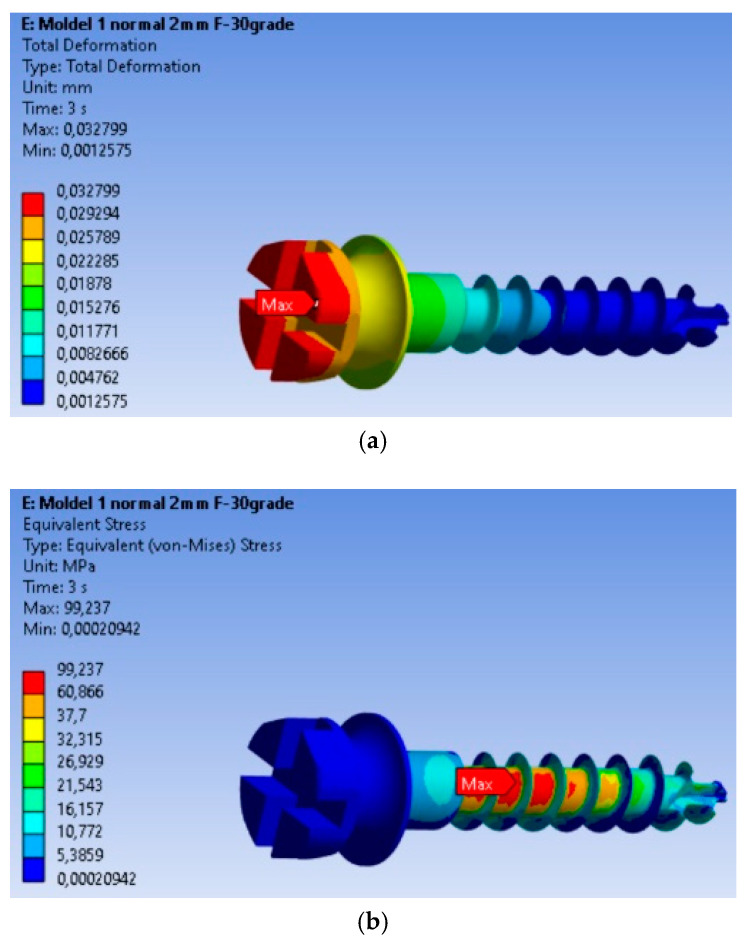

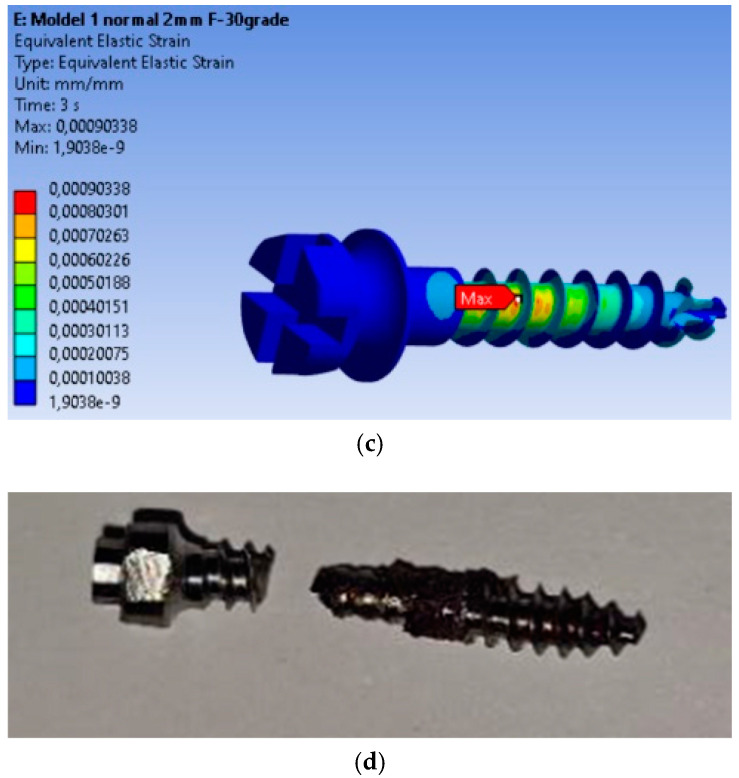
Results for the mini-implant under 2 N loading at 30°: (**a**) Total deformations; (**b**) von Mises equivalent stresses; (**c**) Equivalent strains; (**d**) The mini-implant was fractured after removal precisely in the area where the FEM analysis indicated the maximum von Mises stress.

The finite element modeling (FEM) analysis provided key insights into the mechanical behavior of the MI under orthodontic loading conditions, focusing on total displacement, von Mises stresses, and equivalent strains. Total displacement analysis (Figure 2a) shows that maximum displacement is 0.032799 mm, occurring at the free end of the MI, where orthodontic forces are applied. This behavior is expected, as the threaded section is fixed in the material, and the bending force results in the largest displacement at the free end. The von Mises stress analysis (Figure 2b) shows that the maximum stress is 99.237 MPa, located in the threaded area, consistent with typical failure points of MIs. The high stress in this region indicates a high likelihood of material fatigue and failure under loading. Equivalent strain analysis (Figure 2c) shows that the maximum strain is 0.00090338, also concentrated in the threaded area. The correlation of stress and strain in this region highlights its susceptibility to mechanical degradation under prolonged or repetitive loading.

The real-world implications of this analysis are evident in Figure 2d, which shows a fractured MI that failed at the exact location where the FEM analysis predicted the highest stress concentration. This physical fracture mirrors the computational predictions, providing validation for the FEM model and confirming the accuracy of the stress and strain distributions obtained.

### 3.2. Safety Analysis of the MI: Safety Factors, Safety Margin, and Stress Ratio

**Figure 3:**
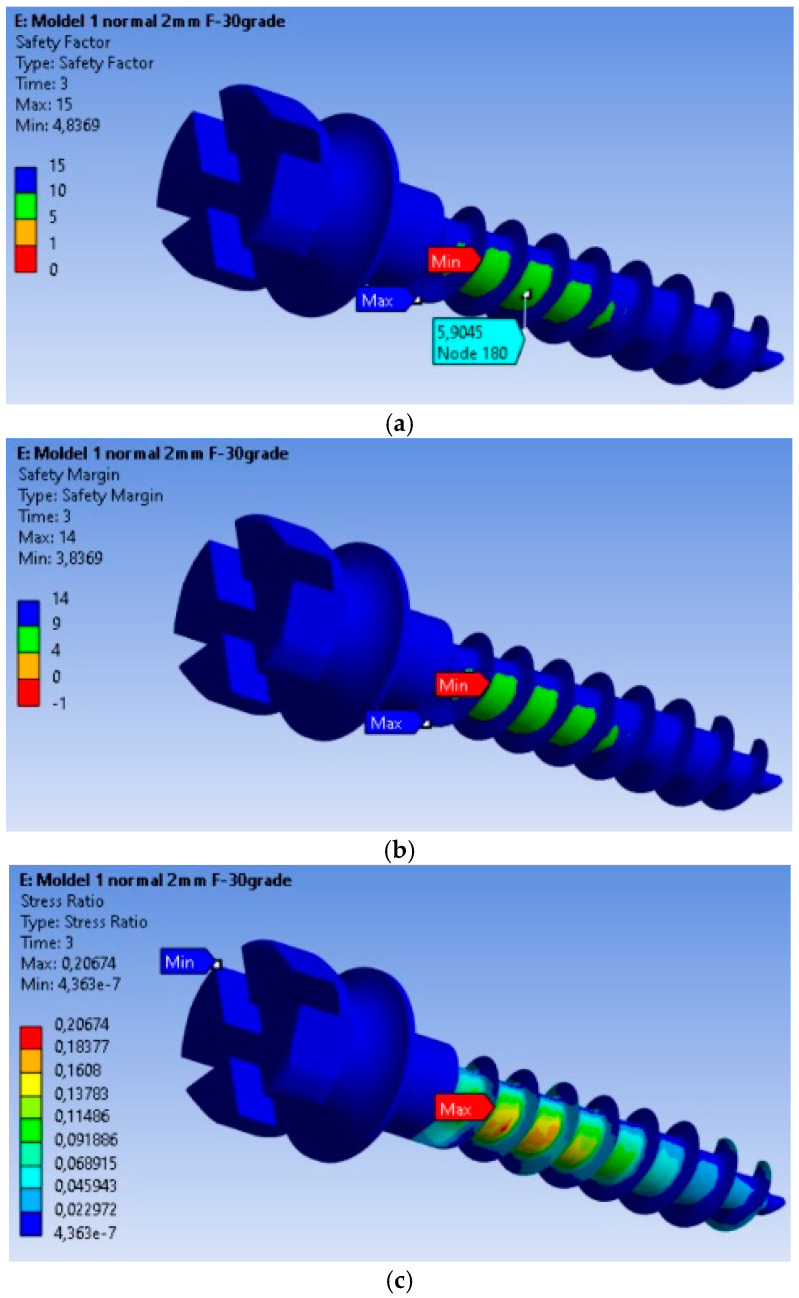
Safety factors obtained for the mini-implant under an applied force of 2 N: (**a**) Safety factors σc/σMax; (**b**) Safety margin (σc/σMax) − 1; (**c**) Stress ratio σMax/σc.

The safety factors obtained for the MI are presented in Figure 3a. The minimum safety factor is 4.8369, located at the most highly stressed node. Therefore, it can be stated that the MI fully complies with the strength requirements. This value is calculated for an orthodontic force of 2 N, and the safety factor is the ratio between the material’s yield strength and the von Mises equivalent stress. The yield strength value was determined experimentally on a titanium MI, with a yield strength of 480 MPa. The safety margin obtained for the MI is shown in Figure 3b, with the minimum safety factor being 3.8369.

In Figure 3c, the stress ratio (calculated stress value/yield strength of the material) is depicted. From this figure, we can observe that the maximum stress ratio is 0.20674, located at the node with the maximum equivalent stress value.

### 3.3. Fatigue Analysis of the MI Under 2 N Orthodontic Force: Life Expectancy, Safety Factors, and Failure Assessment

**Figure 4:**
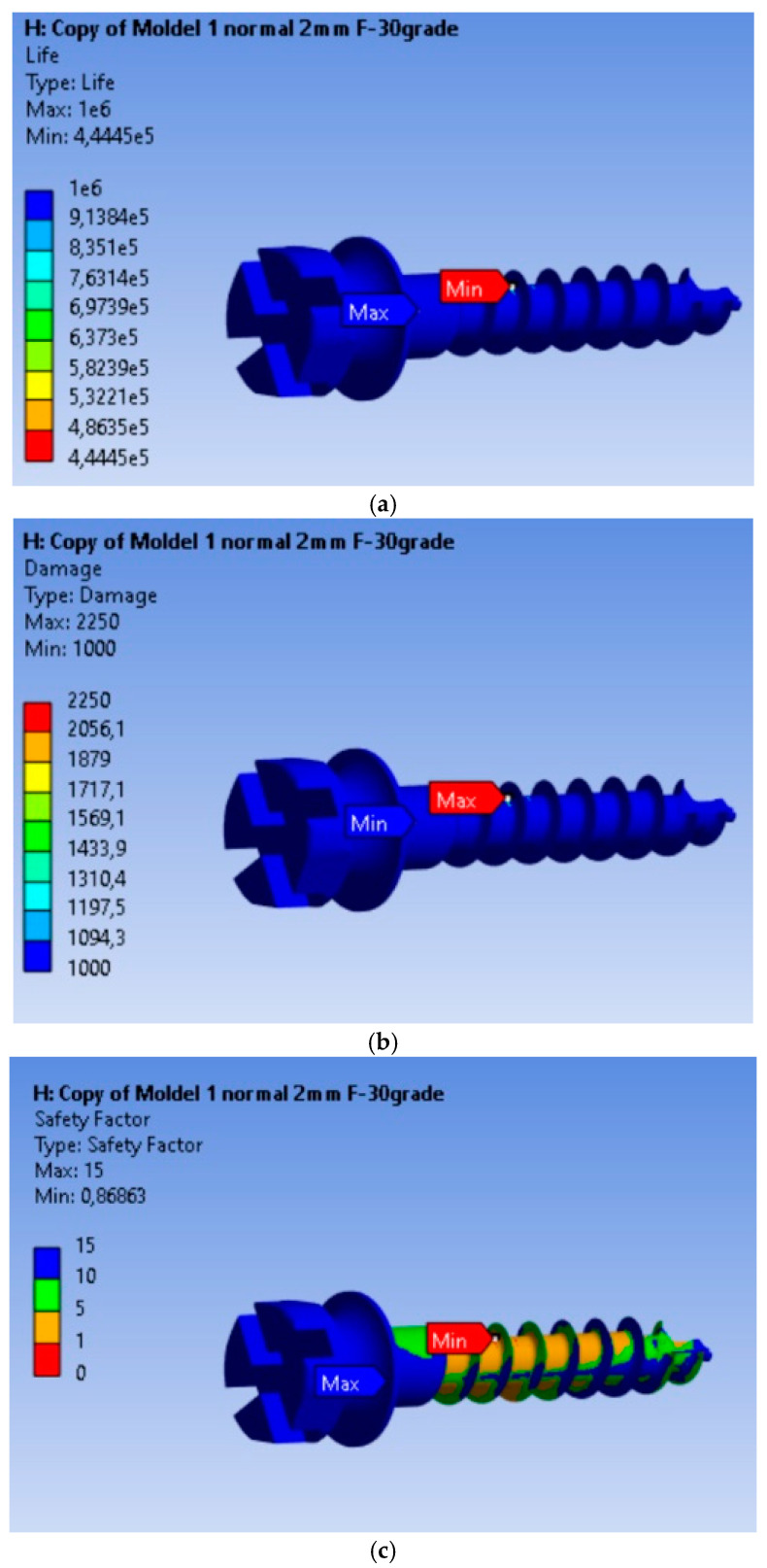
Results obtained for mini-implant endurance under a force of 2 N: (**a**) Endurance; (**b**) Failure; (**c**) Fatigue safety factors.

The fatigue calculation was performed for a symmetric alternating regime, following the ASME elliptical theory [23]. The type of analysis was life expectancy according to the equivalent stress criterion recorded based on the von Mises criterion, with a scaling factor of 1. Based on the results obtained, it is found that the MI will operate without defects for a duration of 4.445 × 10^5^ cycles under an orthodontic force of 2 N. From Figure 4a–c, we can observe that this minimum is achieved for a very small volume of material, and the life expectancy values for the remaining material are 10^6^ cycles. The maximum of 2250 is located at the node for which the minimum fatigue life value was calculated, being 2.5 times greater than in the rest of the material. The fatigue safety factors obtained for the MI are presented in Figure 4c. The minimum fatigue safety factor is 0.86863, located at the most highly stressed node.

### 3.4. Fatigue Performance of the MI Under 10 N Orthodontic Force: Life Expectancy, Safety Factors, and Failure Analysis

**Figure 5:**
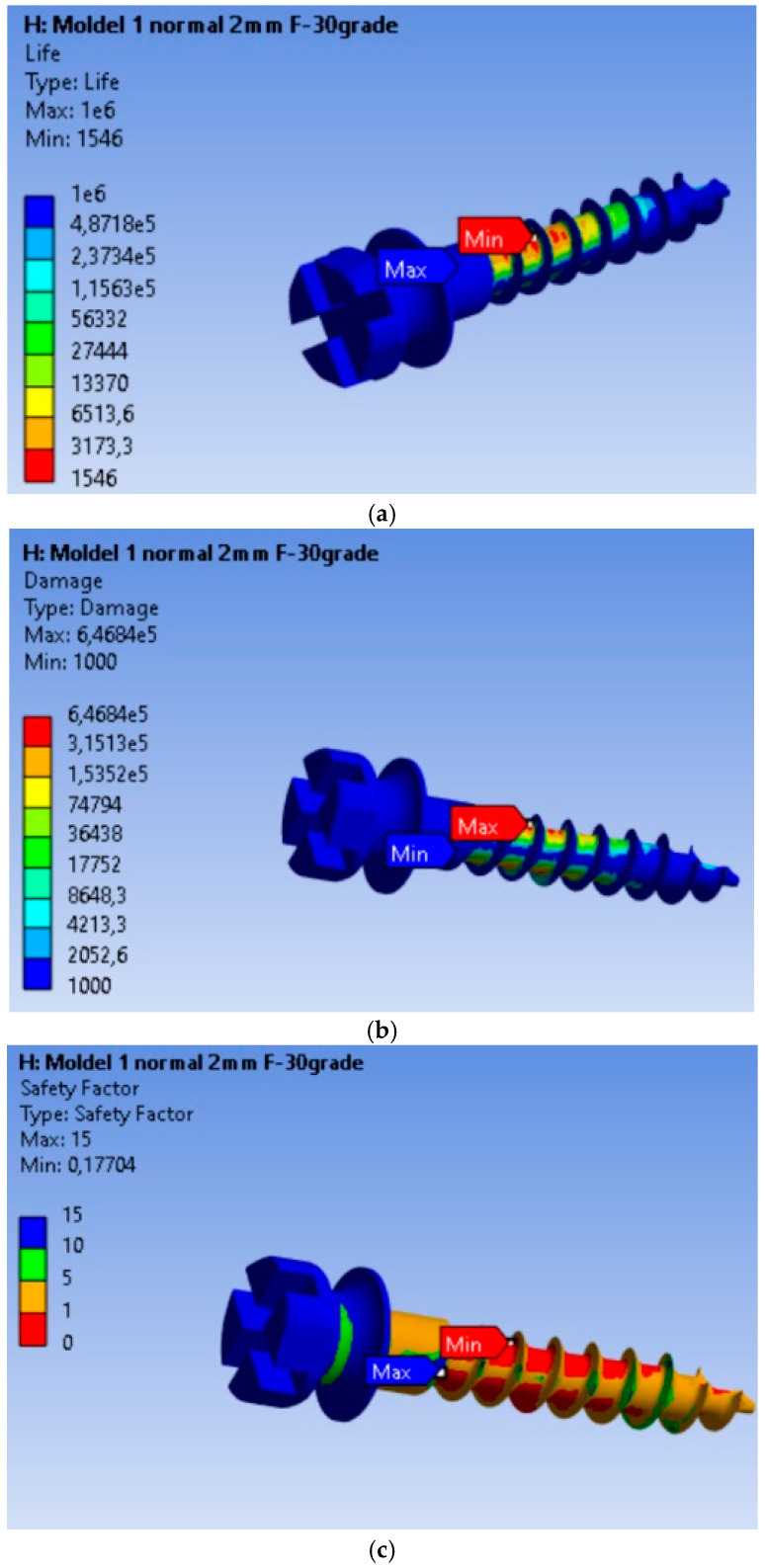
Results obtained for mini-implant endurance under a force of 10 N: (**a**) Endurance; (**b**) Failure; (**c**) Fatigue safety factors.

The endurance or operating time under safe fatigue loading conditions for the scenario where the orthodontic force is 10 N is shown in Figure 5a. Based on the results obtained, it is found that the MI will operate without defects for a duration of 1546 cycles. This value is 287.48 times lower than in the case of the 2 N load. Thus, for an orthodontic force of 10 N, the MI will last without defects for a much shorter period compared to a 2 N load. Failure is shown in Figure 5b, where the maximum value of 6.468 × 10^5^, located at the node with the minimum fatigue life, is 6.468 × 10^2^ times greater than in the rest of the material. The fatigue safety factors obtained for the MI are presented in Figure 5c. The minimum fatigue safety factor is 0.17704, located at the most highly stressed node.

Compared to the 2 N loading, the 10 N (Table 3) load results in a significantly shorter lifespan, affects a larger volume of material, and reduces the fatigue safety factor, suggesting that the MI is much more vulnerable to structural failure under higher loads. This highlights the importance of maintaining an optimal force in orthodontic treatments to prolong the lifespan of implants.

## 4. Discussion

The tolerance limits for deformations in orthodontic MIs are critical for ensuring their effectiveness and longevity during orthodontic treatment. Understanding the factors influencing these tolerances is essential for optimizing their clinical application.

Applying a 2 N loading force on orthodontic MIs is an important factor in orthodontic treatment, as it directly impacts the stability and efficiency of the MI during tooth movement. Research indicates that a loading force of 2 N falls within the clinically accepted range for immediate loading of MIs, which is essential for ensuring effective anchorage during orthodontic procedures [4]. This force level is often utilized in clinical settings for various orthodontic applications, including canine retraction, where forces of 1.5 N to 2.5 N are commonly recommended [24]. The biomechanical effects of applying a 2 N force on MIs have been thoroughly investigated. The application of a 2 N loading force on orthodontic MIs is supported by clinical evidence as being within safe limits for immediate loading. Finite element analyses have revealed that the stress distribution around MIs is heavily affected by both the magnitude of the applied force and the properties of the surrounding bone [25]. It is well-established that MIs can tolerate immediate loading; however, excessive forces may result in complications such as implant displacement or failure [26].

Finite element analysis (FEA) is a highly effective computational method for predicting the mechanical behavior of structures under different loading scenarios. However, the accuracy of FEA results is highly dependent on the assumptions made during modeling. The presumption of 100% osseointegration simplifies the model but neglects the variability inherent in biological systems. This can lead to erroneous predictions regarding stress distribution, load transfer, and potential failure modes of implants [27].

### 4.1. Discussions on Displacement, Stress, Strain, and Safety Factors in Mini-Implants

In terms of displacement analysis, the maximum total displacement of 0.032799 mm observed at the end of the MI highlights a significant stress concentration in this area. This finding can be utilized to assess the degree of bending and deformation the MI endures during orthodontic treatment.

The von Mises stress analysis revealed a maximum stress of 99.237 MPa in the threaded region, indicating a high-stress concentration that could signify the critical failure point of the MI. This finding suggests that discussions should focus on design and manufacturing strategies to enhance the implant’s ability to withstand these stress levels and reduce the risk of failure during use.

Equivalent linear strains: The maximum values of equivalent linear strains, such as 0.00090338 MPa in the threaded area, are important for evaluating the degree of deformation of the MI material. These data can be used to improve the design and materials used in MIs to minimize deformation and improve stability during use.

Interpretation of safety factors: The minimum safety factor value indicates that the MI meets the established strength requirements. This indicates that the MI possesses sufficient strength to endure the applied orthodontic forces, minimizing the likelihood of deformation or fracture.

The stress ratio, which is the ratio of the calculated stress to the material’s yield strength, provides critical insight into the loading conditions of the MI relative to its maximum strength capacity. This metric is essential for evaluating the safety margin and ensuring that the applied forces remain within the implant’s structural limits. The maximum stress ratio indicates significant stress, and its location at the nodes with the maximum equivalent stress value can be considered a critical point regarding the stability and integrity of the MI.

The fatigue analysis revealed that failure is localized in a specific node of the MI, where the minimum fatigue life was recorded. This suggests a potential point of vulnerability in the structure of the MI and can serve as a focal point for future design and manufacturing improvements. The estimated lifespan of approximately 4.445 × 10^5^ cycles is an indication of the reliability and stability of the MI under repeated loads. The analysis indicates that the MI is much more susceptible to damage under a higher load, which may impose restrictions on the application of greater orthodontic forces during treatment. Failure is localized in a specific node of the MI, where the fatigue life is minimized, and the fatigue safety factors indicate a significant decrease in the device’s reliability under this higher load.

These results emphasize the importance of adapting orthodontic treatment to the capabilities and limitations of each MI and the need to consider the forces applied to prevent damage and failure of the device.

Nienkemper et al. emphasized that loading duration is a critical factor affecting MI displacement under orthodontic loading, suggesting that the mechanical behavior of MIs is not solely dependent on the force magnitude but also on the duration of the applied forces [26]. Research indicates that MIs are designed to withstand certain force thresholds, with optimal loading typically recommended to be around 3.75–4.5 N [25].

### 4.2. Future Research Directions

Based on these results, future research directions could be suggested to further investigate the mechanical behavior of MIs and develop new strategies or technologies to improve their stability and performance in clinical practice. This could include additional computer simulation studies or experimental tests to validate and expand upon the current findings.

### 4.3. Limitations and Future Directions

(i) Use of a single implant length (12 mm): An important aspect of this study is that only MIs with a length of 12 mm were used. Future studies should include a wider range of lengths to better understand the relationship between MI length and mechanical performance;
(ii) Finite models of the bone: The bone modeling was done using linear, elastic, and isotropic mechanical properties. This simplification does not accurately reflect the complex and heterogeneous behavior of real bone, particularly cortical and spongy bone;
(iii) Use of only titanium MIs (Ti6Al4V): As a result, the study does not offer a comparison between the various available materials, such as 316L, which could influence the clinical performance and long-term stability of MIs.
(iv) Ideal insertion and loading conditions: The study assumed that MI insertion was performed under ideal conditions and that complete osseointegration (100%) was achieved. In clinical practice, insertion conditions vary, and osseointegration may not always be complete, which could affect the performance of implants.

## 5. Conclusions

The results of this study highlight the critical importance of maintaining orthodontic forces within an optimal range, approximately 2 N, to preserve the structural integrity and functional longevity of MIs in clinical applications. Finite element analysis indicates that under a 2 N load, the MI can sustain approximately 445,000 cycles with stress and strain levels that remain within safe limits for both cortical and spongy bone. In contrast, a 10 N load leads to a drastic reduction in implant lifespan, estimated at only 1546 cycles, along with significantly elevated stress and strain concentrations. The pronounced increase in mechanical load at 10 N introduces heightened risks of implant failure and potential damage to the surrounding bone, particularly in the cortical bone adjacent to the anchorage point.

These findings underscore the necessity of carefully calibrating orthodontic forces to enhance MI stability and minimize adverse effects on bone health. By adhering to controlled force parameters, clinicians can optimize treatment outcomes, extending the lifespan of MIs and safeguarding peri-implant bone integrity.

## Figures and Tables

**Table 1:** The material properties utilized in the finite element analysis were modeled based on specific characteristics to ensure accuracy and realism [22].

Material/Component	Elastic Modulus (MPa)		Poisson’s Ratio
Bracket	380,000		0.19
Mini-Implant	110,000	200,000	0.3
Tooth	84,100		0.2
PDL	68.9		0.45
Cortical bone	17,000		0.3
Spongy bone	350		0.25

**Table 2:** Finite element analysis model parameters.

FEA Parameter	Property
Property discretization (nodes/elements)	356,422/229,672
Element	10 node tetrahedron
Software	ANSYS Workbench 19.2, Canonsburg, PA, USA
Material model	Isotropic, homogeneous, linear
Contact model	Friction (nonlinear, friction coefficient = 0 between mini-implant and bones), bonded (linear)
Loading	Oblique (0.1–10 N) (30°, 45°, 60°)
Mini-implant type	Threaded, diameter: 2.0 mm, 12.0 mm length
Boundary conditions	100% osseointegration

**Table 3:** Comparison of 2 N and 10 N force effects on the mini-implant.

Parameter	2 N Force	10 N Force
Endurance (operating cycles)	4.445 × 10^5^ cycles	1546 cycles (287.48 times lower)
Volume of material affected	Small volume of material	Larger volume of material
Minimum fatigue safety factor	0.86863 (located at most stressed node)	0.17704 (located at most stressed node)
Maximum stress concentration	2250 MPa (2.5× higher than the rest of the material)	6.468 × 10^5^ MPa (646.8× higher than the rest of the material)

## Data Availability

Data are contained within the article.
